# Evaluating the impact of decentralising tuberculosis microscopy services to rural township hospitals in gansu province, china

**DOI:** 10.1186/1471-2458-11-103

**Published:** 2011-02-15

**Authors:** Xiaolin Wei, Guanyang Zou, Hui Zhang, Renzhong Li, John D Walley, Shiwen Jiang, Jia Yin, Shuigao Jin, You Li, Qiang Sun, James N Newell, Sian Griffiths, Lixia Wang

**Affiliations:** 1 School of Public Health and Primary Care, Chinese University of Hong Kong, 2/F School of Public Health, Prince of Wales Hospital, Shatin, NT, Hong Kong, China; 2 China Office, Nuffield Centre for International Health and Development, University of Leeds, Leeds, UK, Rm 1220, 1032 Dong Men Bei Lu, Shenzhen, 518003, China; 3 China National Centre for Tuberculosis Prevention and Control, China Centre for Disease Control, 155 Changbai Road, Chang Ping District, Beijing, 102206, China; 4 Nuffield Centre for International Health and Development, University of Leeds, Leeds, UK, 101 Clarendon Rd, Leeds, LS2 9LJ, UK; 5 Chief Scientist, China Centre for Disease Control, Address: 155 Changbai Road, Chang Ping District, Beijing, 102206, China; 6 School of Public Health, and Centre for Health Management and Policy, Shandong University, 44, Wenhua Xi Lu, Jinan, 250012, China

## Abstract

**Background:**

In 2004, the Ministry of Health issued the policy of decentralising microscopy services (MCs) to one third of all township hospitals in China. The study was conducted in Gansu Province, a poor western one in China. Ganzhou was one county in Gansu Province. Ganzhou County was identified as a unique case of further decentralisation of tuberculosis (TB) treatment services in township hospitals. The study evaluated the impact of the MC policy on providers and patients in Gansu Province. The second objective was to assess the unique case of Ganzhou County compared with other counties in the province.

**Methods:**

Both quantitative and qualitative methods were used. All 523 MCs in the province completed an institutional survey regarding their performance. Four counties were selected for in-depth investigation, where 169 TB suspects were randomly selected from the MC and county TB dispensary registers for questionnaire surveys. Informant interviews were conducted with 38 health staff at the township and county levels in the four counties.

**Results:**

Gansu established MCs in 39% of its township hospitals. From January 2006 to June 2007, 8% of MCs identified more than 10 TB sputum smear positive patients while 54% did not find any. MCs identified 1546 TB sputum smear positive patients, accounting for 9% of the total in the province. The throughputs of MCs in Ganzhou County were eight times of those in other counties. Interviews identified several barriers to implement the MC policy, such as inadequate health financing, low laboratory capacity, lack of human resources, poor treatment and management capacities, and lack of supervisions from county TB dispensaries.

**Conclusion:**

Microscopy centre throughputs were generally low in Gansu Province, and the contribution of MCs to TB case detection was insignificant taking account the number of MCs established. As a unique case of full decentralisation of TB service, Ganzhou County presented better results. However, standards and quality of TB care needed to be improved. The MC policy needs to be reviewed in light of evidence from this study.

## Background

China had 1.8 million tuberculosis (TB) patients ranking the second in the world [1] The National TB Control Programme was implemented in the 1990 s. It was based on the internationally-recommended World Health Organization (WHO) strategy known as DOTS. DOTS coverage reached 100% in China in 2005 [1]. However, TB control remains a challenge, particularly in the rural western provinces. Western provinces had 70% higher of TB prevalence than eastern provinces [2]. Sputum smear microscopy is recommended by the DOTS programme as the most cost-effective way for TB diagnosis [3]. However, access to qualified microscopy service was limited in many high TB burden countries [1,4], including western China [5].

China's rural health service is a three-tier system. The primary tier is village clinics, which provide basic acute and preventative care. The tertiary tier is county hospitals, which provide specialised outpatient and inpatient care. Township hospitals are the secondary tier. It is responsible for providing public health services, ambulatory and basic inpatient care for a population ranging from 50,000 to 150,000. 'Central township hospitals' are better equipped and staffed ones. They provide technical support to surrounding township hospitals. China's western rural areas were poorly financed and had unfavourable health indicators compared with their eastern counterparts [6].

TB control in China mainly relies on a centralised management system. Symptomatic TB patients visiting any kind of health facility should be referred to the county TB dispensary (CTD), where free microscopy service is provided, followed by free treatment of WHO DOTS chemotherapy [7]. Other health providers should not treat TB patients. Township hospital and village doctors have the responsibility of supervising TB patients during their treatment. Previous studies have identified barriers of rural patients in accessing TB care, such as limited staff capacity in CTDs, poor access from villages to CTDs, and high costs of TB treatments [5,8-12].

In 2004, a national policy was introduced to decentralise microscopy services into one third of all township hospitals in China. The Ministry of Health issued the notice to establish microscopy centres (MC) at township hospitals. This policy aims to *1) improve access of patients to sputum tests in rural areas; and 2) improve the case detection rate of new sputum positive cases *[13]. The MC was not a standing alone organisation. It was virtually established in the laboratory of the township hospital. Technicians in the laboratory were trained for sputum smear services, while the outpatient doctors in the township hospital were responsible to identify TB suspects and refer them to microscopy service in its laboratory, or the MC. According to the policy, positive cases identified from the MCs should be sent to the CTD for confirmation within seven days. Patients suggestive of TB should be referred to the CTD for further diagnosis. The Ministry of Health's notice required that the laboratory of the MC should have adequate bio safety risk protection similar to that of the CTD [14].

The study aimed to evaluate MC performance and its impact on patients in Gansu Province. The province is covered by mountains and Gobi deserts, with very little arable lands. In 2007, its total population was 26 million, of whom 68% lived in rural areas. Its population density was 58 per square kilometer, substantially lower than the national average of 134. Gansu was the second poorest province in China, as its gross domestic products (GDP) per capita was only half of the national average [15].

Gansu Province has 87 counties/districts, and Ganzhou County is one of them. During our fieldwork, Ganzhou County was found as a unique site where the county health bureau decided to further decentralise TB diagnosis and treatment care to township hospitals with MCs. Thus, Ganzhou County was a special case. The second aim of the study was to compare the MC performance in Ganzhou County with other counties.

## Methods

A multi-method study design was employed. This included (1) institutional surveys of all MCs in Gansu Province, (2) patient surveys in four counties in the province and (3) key informant interviews with service providers. Data collection was conducted from July to December, 2008. Quantitative data was collected by staff of the Gansu Provincial Centres for Disease Control, while qualitative data was collected by the staff of the National Centre for TB Control. All data collectors attended a one-day training workshop. Informed consent was obtained from all participants. Ethical approval for the study was obtained from the Research Ethics Committee of the China National Centre for TB Control.

### Institutional survey

All 87 county TB dispensaries in the province responded to a structured institutional survey. Questions included socio-economic characters of the population covered by the MC and the county, distance of the MC to the CTD, the number of TB suspects examined and smear-positive cases identified by MCs and CTDs, frequencies of training and supervisions provided to MCs.

### Patient questionnaire survey

Four counties were purposively selected by the provincial TB programme. Two counties (Ganzhou and Shandan) were located in plain and Gobi with relatively high income, while the other two (Xifeng and Zhenyuan) were located in mountains with relatively low income. Background information about the selected counties is presented in Table 1.

**Table 1:** General information of selected counties for patient survey

	Ganzhou	Shandan	Xifeng	Zhenyuan
Geography	Plain & Gobi	Plain & Gobi	Mountain	Mountain
Population	506,558	192,947	330,000	520,000
Per capital income (RMB)	3,947	2,892	1,734	1,500
Registered active TB patients in 2006	2,836	1,295	7,66	1,036
Registered smear positive patients identified in 2006	295	110	200	216

A structured questionnaire (Additional file 1) was developed for the patient survey asking questions of TB suspects who presented or were referred to the MC or TB dispensary due to chronic cough. TB suspects here were defined as patients who had persistent cough for more than two weeks [7,16]. Questionnaires were developed based on a previous similar study [17] and revised by a national team. Questions included demographic information, social-economic status, knowledge regarding MCs, costs and delays to TB diagnosis. Forty TB suspects were randomly selected from each county based on the laboratory registers of TB suspects who had a sputum examination from July 2007 to April 2008. In each county, 20 TB suspects were randomly selected from two township hospitals equipped with MCs. The other 20 TB suspects from the same townships but not having a sputum examination record in the township hospitals were randomly selected from the CTD laboratory registers. In total, 169 patients participated in the questionnaire survey.

### Interviews

Key informant interviews were conducted in the four counties covering operational details of the MCs, including financing, human resources, diagnosis and treatment. We interviewed the CTD directors and laboratory technicians, township hospital directors, outpatient doctors and laboratory technicians. TB directors and laboratory technicians at the prefecture and provincial levels were also interviewed. In total, 38 interviews were conducted.

### Data analysis

The quantitative data was checked, coded and double entered into a database using Microsoft Access and then analyzed using SPSS 14.0 (SPSS Inc, Chicago, USA). Descriptive analysis of mean and median was conducted. Independent t-test, chi-square test, one-way ANOVA and Mann Whitney rank tests were used when appropriate. A linear regression model was conducted to identify factors correlated with the number of TB suspects examined in an MC as the dependant variable. Independent variables included variables from the institutional survey that having a potential theoretical relationship with the dependent variable, i.e., population covered by the MC, per capita income in the MC catchments area, whether the MC received supervision visits from the CTD, and distance from the MC to the CTD.

Qualitative data was audio taped and pencil noted. Transcripts were input into computer and checked by supervisors. Two researchers independently developed the codes, and then codes were compiled together to form an analytical frame with consensus from the research team. Thematic framework analysis was employed to guide the analysis. That is, key themes that emerged frequently from the codes were identified with interpretations. Then themes were combined based on their interpretations into bigger key themes according to their nature and policy relevance.

## Results

### Decentralisation of microscopy service in Gansu Province

By June 2007, 523 MCs had been established in all the 87 counties/districts in Gansu Province. Of the total 1347 townships in Gansu, 39% were equipped with MCs. From January 2006 to June 2007, 74 (14%) MCs did not examine any TB suspects and 279 (54%) MCs did not find any smear-positive patients. Only 44 (8%) of MCs identified more than 10 smear-positive patients. In total, 27,959 TB suspects were examined and 1,546 smear-positive patients were identified in MCs. These accounted for 21% of total TB suspects examined in the province and 9% of total smear positive cases identified in the province. On average, each MC checked 54 TB suspects and identified 3 smear-positive patients (Table 2).

**Table 2:** Performance of CTD and MC from 2006 to Jun 2007

	CTD	MC	Total
Numbers of centres	87	523	
TB suspects tested			
Number (%)	108,202 (80)	27,959 (21)	136,959
Mean per site	1244	54	
Median per site	1019	32	
TB smear positive cases identified			
Sum (%)	15,863(91)	1,546(9)	17,409
Mean	182	3	
Median	154	0	

CTD: county TB dispensary; MC: Microscopy centre

MCs covering a population of more than 30,000 had a higher average number of TB suspects examined per MC than other groups (P < 0.001, Table 3). Counties with lower per capita income, i.e., with GDP per capita between RMB 1000-2000 (US 147-294), examined more TB suspects per MC than their richer counterparts (P < 0.01). MCs who received supervision visits from CTDs had more TB suspects examined than those not receiving any supervision (P = 0.013). The multi variable regression analysis identified that more TB suspects were examined per MC in poorer areas (β = -0.088, P < 0.001), and in MCs covering a larger population (β = 0.162, P = 0.001).

**Table 3:** Number of TB suspects checked per MC in association with MC's service population,

Indicators		No. of TB suspects checked Mean (Median)
Service population	≤10000	39 (18)
	10001-20000	45 (25)
	20001-30000	54 (34)
	>30000	69 (48)^a^

Per capita income of MC covered areas:		
		
	≤1000 RMB(USD 147)	42 (24)
	1001-2000 RMB (USD 147-294)	65 (43)^b^
	2001-3000 RMB(USD 294-441)	40 (23)
	>3000 RMB(USD 441)	39 (22)

Supervision visit from CTD	Yes	55 (34)^c^
	No	47 (21)

a Significant differences was found among groups (F = 8.232, χ^2 ^= 12.637, P < 0.001). Group ' > 30,000 population was significantly higher than other groups (P < 0.05).

b Significant difference was found among groups. '1001-2000 RMB(USD 147-294)' had higher number than the two higher income groups (P < 0.01).

c MCs with supervision checked more TB suspects (Mann-Whitney test, Z = -2.476, P = 0.013)

Of all surveyed TB suspects, 121 (71.6%) visited the township hospitals with MCs while 48 (28.4%) did not. MC visitors were older (P = 0.008) and had a lower level of education (P < 0.001), but they had better knowledge of the availability of microscopy service than non MC visitors (P < 0.001). In general, all TB suspects reported shorter distance to travel (Z = -14.3, P < 0.001), less travel time (Z = -12.4, P < 0.001) and less transportation cost (Z = -14.1, P < 0.001) from home to the township hospital than to the CTD (Table 4).

**Table 4:** Socio-economic and transportation information of TB suspect surveyed

	TB suspects who visited MCs	TB suspects who did not visit MCs	TB suspects of Ganzhou residents	TB suspects from the other three counties	Total
Number (%)	121 (72)	48 (28)	42 (25)	127 (75)	169
Age (mean)	57^a^	50	62^a^	52	56
Male (%)	82 (68)	32 (67)	29 (69)	85 (67)	114 (68)
Education of primary school and below (%)	88 (73)	18 (38)^b^	36 (86)	70 (55)^b^	106 (63)
Married (%)	98 (81)	41 (85)	38 (91)	101 (80)	139 (82)
Farmer (%)	92 (76)	36 (75)	40 (95)	88 (69)	128 (76)
Geography: Plain	87 (72)	39 (71)	42 (100)	78 (61)	153 (91)
Mountain	34 (28)	16 (29)	0	49 (39)	16 (10)
Number of family members (mean)	4.1	4.4	3.8	4.4	4.2
If having rural health insurance (%)	103 (85)	44 (92)	42(100)	105 (83)	147 (87)
If knowing the availability of microscopy service in MCs (%)	74 (61)^c^	16 (33)	23(55)	67 (53)	90 (53)
Distance from home to the CTD (km)	29	31	13	35	30^d^
Distance from home to the MC (km)	4.9	8.1	4.9	6.0	5.7
Travel time to the CTD (min)	140	143	103	153	141^d^
Travel time to the MC (min)	48	57	55	49	51
Travel costs to the CTD(RMB)	13	15	6	16	13.5^d^
Travel costs to the MC (RMB)	1.7	2.6	1.5	2.1	1.9

a TB suspects visiting MC were older than those not visiting MC (Z = -2.794, P = 0.005); TB suspects of Ganzhou were older than those of other counties (Z = -3.341, P = 0.01)

b TB suspects visiting MCs were significantly higher than those of not visiting MCs (χ^2 ^= 18.240, P < 0.001); TB suspects of Ganzhou were significantly higher than others (χ^2 ^= 12.637, P < 0.001)

c TB suspects visiting MC were significantly higher than those not visiting MCs (χ^2 ^= 10.687, P = 0.001).

d TB suspects' travel distance was significantly shorter to the MC than the CTD, (Z = -14.263, P < 0.001), while accompanied with significantly shorter travel time (Z = -12.356, P < 0.001) and less travel cost (Z = -14.153, P < 0.001).

Qualitative analysis identified two emerging themes: MC capacity and financing of the microscopy service decentralisation in Gansu Province.

#### MC capacity

Inadequate infrastructure of MCs in the township hospitals was identified as a major barrier to decentralise microscopy services. It posed concerns for bio safety risks. Interviews showed that sputum smearing was conducted in the same area with other laboratory tests in many MCs. The majority of MCs we visited had not installed ventilation cabinets for sputum smearing. The provincial laboratory technician said, *"In most of MCs, sputum tests are conducted in the corner of the laboratory, or in the toilette. There is only a water basin and a faucet. Nothing is available for personal protection. No extra professional equipment's available for disinfection. There is problem of inadequate awareness of personal protection. But I think the problem mainly comes from lack of funding."*

In Gansu Province, all positive smears were sent to the CTD for double checking. This created enormous workloads for CTD laboratories. On the other hand, the workload of MC laboratory technicians was low. The majority of MC technicians were part-time with high turnover rate. Most spent two hours per week on sputum tests, examining 6-8 slides per month. There was not enough on-site laboratory supervision from the CTD to MCs due to capacity limits in the CTD. MC performance largely relied on the referral of TB suspects from outpatient doctors of the township hospital. However, many outpatient doctors lacked the knowledge of the definition of TB suspects and did not how to detect them.

#### Finance

Lack of funding was identified as the major problem in all township hospitals equipped with MCs. Although the Ministry of Health finding for MC was available, the province and local government were not able to provide a 1:1 match fund. In practice, basic laboratory consumables were covered, but there was no fund to cover the costs of human resources and MC maintenance. As a result, township hospitals had to rely on other ways to cover the costs, such as treating patients. One director said, *"We have 25 staff with only 7 funded by the government. We have to earn benefits from patient out-of-pocket costs to support our staff and the MC!" *In several township hospitals, TB suspects were first referred for X-ray examination because the services need to be paid out-of-pocket. Free sputum tests were only undertaken after a shadow was found in the X-ray. We found that many TB suspects were directly referred to the county TB dispensary without being examined sputum locally. One possible reasons was that doctors received cash incentives for referring TB patients to the CTD while no incentives were given to refer internally.

### Ganzhou County: a case of further decentralisation of TB care in the province

Ganzhou was identified during the research as a unique case on full TB service decentralisation. The decision was made by the local health bureau on the basis that the CTD did not have enough capacity to provide TB services. The Ganzhou County government provided specific fund to set up MCs in 10 out of its total 22 township hospitals. Sputum smear positive TB patients were diagnosed in the MCs, while slides were sent to CTDs for confirmation within two days. Then TB patients were treated by the township hospitals with MCs for the whole course of DOTS programme.

The institutional survey showed that MCs in Ganzhou County examined significantly higher numbers of TB suspects and detected more smear-positive patients compared with their counterparts in the province (P < 0.001). On average, each MC in Ganzhou County examined 118 TB suspects and identified 6.4 smear-positive patients, compared to 15 and 0.8 respectively in their counterparts. Ganzhou MCs examined 35% of TB suspects and identified 27% of smear-positive patients in the county, compared with the average of 21% and 9% in the province, respectively.

Of all surveyed TB suspects, 42 (25%) were Ganzhou County residents and 127 (75%) were from other counties (Table 4). Higher proportions of Ganzhou residents visited MCs (93%) compared with their peers in other counties (65%, P = 0.004). 65% of TB suspects visiting township hospitals in Ganzhou were then diagnosed and treated there. On average, TB suspects from Ganzhou spent less on medical costs before diagnosis (USD 13 v.s. USD 70; P = 0.028) and less travel costs (USD 1 v.s. USD 5, P < 0.001) compared with those from other counties.

Qualitiative results showed that MC laboratory staff in Ganzhou County had a higher workload on sputum examination than their counterparts in other counties. Despite the improved financial situation, infrastructure in Ganzhou was still below the Ministry of Health requirements. In an MC, service decentralisation was regarded as a way to attract patients, as the hospital director commented, *"We were not on the MC list initially. But we applied to be one for better reputation. MCs brought patients indirectly to us. We can treat complications and even hospitalize patients"*. A number of suboptimal TB services were identified in Ganzhou township hospitals. The misuse of antibiotics were common for chronic cough patients. Many sputum positive patients were observed being prescribed anti-TB drugs along with broad spectrum antibiotics at the same time.

## Discussion

Gansu Province has set up MCs in 39% of its townships, above the 1/3 target set by the Ministry of Health. Overall the throughputs of MCs in Gansu were low. Although 523 microscopy centres were established in the province, over half did not identify any smear-positive patients and only 8% reported more than 10 smear-positive patients. The low throughputs of MCs may indicate an excessive number of MCs in the province. A similar problem was identified in another province implementing the same national policy [17].

Four health system issues were identified as the major barrier to microscopy decentralisation: 1) Local governments in Gansu Province did not provide adequate training to outpatient doctors and lab technicians. Township hospitals in western China were inadequately funded by the government [11] and they had no financial capacity to invest in MCs. 2) The laboratories for sputum microscopy examination lacked necessary bio safety risk protection, which may pose a threat to their technicians. 3) The perverse incentives were prevalent in township hospitals [18]. Township hospitals had no motivation to provide microscopy services because it was free of charge. The rapidly expanded new rural health insurance had little financial protection for outpatient services [19]. Township hospitals may induce more out-of-pocket expenditure for TB suspects. 4) Training for outpatient doctors in the township was inadequate on how to identify TB suspects and refer them for sputum tests in the MCs. A significant number of TB suspects who visited township hospitals with MCs were not given microscopy examination.

On the positive side, decentralisation had improved the delivery of microscopy service to elders and the poor in Gansu Province. Large number of poor residents tend to use services from township hospitals more often because township hospitals are close to villages and cheaper[8,20,21]. Comparatively speaking, MCs performed better in poor provinces than well-off provinces, despite their general low throughputs [22]. Better MC performance was found in MCs covering a larger population, poorer areas and among TB suspects who knew the service availability. These factors could be taken into account for planning TB services decentralisation.

Ganzhou County was a unique case where TB services were fully decentralised. MC throughputs in Ganzhou County were much higher compared with other counties. Other studies have demonstrated that decentralising a full set of TB services to township hospital may improve rural patients access to TB care, reduce patient costs and improve patient satisfaction [12,23-29]. It is imperative to ensure the quality of care in decentralisation. Decentralisation may attract more patients seeking general healthcare in the township hospitals, hence improving the overall revenue of township hospitals and raising its reputation [12]. However, problems in Ganzhou County also need to be noticed. The decentralisation was a pragmatic decision. No systematic approaches were undertaken. For example, 40% of smear positives cases identified in Ganzhou MCs were referred to the CTD rather than being treated locally. Suboptimal TB care was identified. There is an urgent need to strengthen its quality assurance.

Decentralising TB services to township hospitals fits well with the WHO strategy of engaging all providers in TB care [30]. A strong and well functional CTD is an prerequisite for TB service decentralisation, because the CTD has to take quality assurance responsibilities [12]. Funding should be targeted at strengthening the laboratory capacity, reversing the perverse staff incentives and retaining a competitive workforce. There is a greater need of this in western China where road accessibility is poor. Based on results from this study, policy recommendations were made regarding what services, where and how to decentralise TB service in the needy areas:

**• What services can be decentralised: **This study, together with another study in Guangxi[12], showed that decentralisation should involve TB diagnosis and treatment care in order to improve patient accessibility. The national policy of MC only decentralised limited functions of microscopy screening service, which did not effectively benefit TB suspects, and lead to suboptimal use of limited resources.

**• Where to decentralise**: Poor western provinces should be given a high priority for TB service decentralisation. Decentralisation of service can only be implemented in a limited number of township hospitals where there is a need. Those township hospitals be well equipped and staffed, have a good reputation locally, and cover certain large number of population. Transportation to the township hospitals should be much easier than to the CTD. Both the CTD and the township hospital should have the needs and agree on the organisational change. The CTD should have adequate capacity to maintain frequent monitoring and evaluation of the MC work to achieve a high quality.

**• How to decentralise: **First, training of the national TB control guideline should be provided to all relevant staff in the township hospital. Training of outpatient doctors is especially important as they are the first point of care for TB suspects. Specific and concise TB case management guidelines including communication, treatment algorithm for both positive and negative cases and patients follow-up have to be developed and adequately trained. Quality insurance activities should include external quality control of laboratories, case management monitoring, as well as adequate training and support provided by the CTD.

Selection bias exists as the counties and MCs were purposively chosen. However, no other relevant studies were available to provide estimates to calculate the sample size. Concerns may arise from the comparison between Ganzhou County and other counties. This was because Ganzhou County was identified during the research fieldwork as a unique case. We felt it as an obligation to report it. The provincial wide institutional survey data provided a representative base for the comparison. Patients' recall bias such as medical expenses and travel costs may exist. However, the time gap between the survey and onset of patient illness was short. Due to capacity limits of the research team, laboratory assessments were partially conducted. However, suboptimal quality was witnessed in all research sites.

## Conclusion

Microscopy centre performance was generally low in Gansu Province, and the contribution of MCs to TB case detection was marginal. It indicated that the national policy should be adjusted to consider the existing resources, capacity and local needs in Gansu Province. As a unique case of full decentralisation, Ganzhou County had better MC performance. It provided a more localised service albeit that the quality of service was of concern. Health service strengthening through adequate financing, qualified staff and quality assurance would support successful decentralisation. The national MC policy needs to be reviewed in light of evidence from this study.

## Competing interests

The authors declare that they have no competing interests.

## Authors' contributions

XW, LXW, HZ, RL, SWJ and SGJ designed the research and tools. XW, LXW and GZ oversaw the study and wrote the manuscript. HZ, RL, JW, SWJ, SGJ, QS, JN, SG provided critical comments to improve the manuscript. XW, GZ, JY performed data analysis of the manuscript. All authors read and approved the manuscript.

## Pre-publication history

The pre-publication history for this paper can be accessed here:

http://www.biomedcentral.com/1471-2458/11/103/prepub

## Supplementary Material

Additional File 1 **Questionnaire for suspected pulmonary tuberculosis cases of the study**. As requested by the editor, the patient questionnaire used in this study was translated into English and presented for readers. Use of the questionnaire for research purpose is encouraged. However, any use of it should be noticed to the authors and must be properly cited in any related research products. Click here for file

## References

[B1] WHO WHO Report 2010: Global Tuberculosis Control 2010 Geneva: World Health Organization

[B2] National Technical Steering Group of the Epidemiological Sampling Survey Report on fourth national epidemiological sampling survey of tuberculosis Chinese Journal of Tuberculosis & Respiratory Diseases 2002 25 1 3 7 11953089

[B3] WHO Toman's Tuberculosis: case detection, treatment and monitoring- questions and answers 2004 Geneva: the World Health Organization

[B4] Ayuo PO Diero LO Owino-Ong'or WD Mwangi AW Causes of delay in diagnosis of pulmonary tuberculosis in patients attending a referral hospital in Western Kenya East African Medical Journal 2008 85 6 263 268 18817022 10.4314/eamj.v85i6.9623

[B5] Wang D Liu J Chin D Progress in tuberculosis control and the evolving public-health system in China Lancet 2007 369 691 696 10.1016/S0140-6736(07)60316-X 17321314 PMC7134616

[B6] Tang S Meng Q Chen L Bekedam H Evans T Whitehead M Tackling the challenges to health equity in China The Lancet 2008 372 9648 1493 1501 10.1016/S0140-6736(08)61364-1 PMC7135088 18930531

[B7] Ministry of Health China Tuberculosis Prevention and Control Plan: guideline for programme implementation 2009 2 Beijing, China: Disease Control Department

[B8] Zhang T Tang S Gao J Whitehead M Persistent problems of access to appropriate, affordable TB services in rural China: experiences of different social-economic groups BMC Public Health 2007 7 19 17288593 10.1186/1471-2458-7-19 PMC1805429

[B9] Ministry of Health Disease Control Department General report Social evaluation report of China TB control: 2004-2005 2006 Beijing: China Union Medical University Press 3 76

[B10] Xu B Jiang QW Xiu Y Diwan VK Diagnostic delays in access to tuberculosis care in counties with or without the National Tuberculosis Control Programme in rural China International Journal of Tuberculosis & Lung Disease 2005 9 7 784 790 16013775

[B11] Gong Y Weng Z Chang C China Ministry of Health Social Evaluation report of Xinjiang Autonomous Region China TB Control Program: Social evaluative study report 2004-2005 2006 Beijing: China Union Medical University Press 291 349

[B12] Wei X Liang X Liu F Walley J Dong B Decentralising tuberculosis services from county tuberculosis dispensaries to township hospitals in China: an intervention study International Journal of Tuberculosis & Lung Disease 2008 12 5 538 547 18419890

[B13] Ministry of Health Notice of setting up sputum collection spots at township hospitals 2004 78 Beijing, China: General Office

[B14] Ministry of Health Guidelines to set up the microscopy centres in township hospitals and laboratory operation. Beijing, China 2004

[B15] China Statistical Bureau China statistical yearbook 2009 2010 Beijing: China statistical publishing house

[B16] WHO Management of Tuberculosis Training for Health Facility Staff Detection case of TB 2003 Geneva: the World Health Organization 1 46

[B17] Wei X Li R Zou G Walley J Newell J Liu Z Evaluating the policy of setting up microscopy centres at township hospitals in Shandong China: Experience from patients and providers Health Policy 2010 95 113 121 10.1016/j.healthpol.2009.11.014 20004490

[B18] Yip W Hsiao W The Chinese Health System at a Crossroads Health Affairs 2008 27 2 460 468 10.1377/hlthaff.27.2.460 18332503

[B19] Wagstaff A Lindelow M Jun G Ling X Juncheng Q Extending health insurance to the rural population: an impact evaluation of China's new cooperative medical scheme Journal of Health Economics 2009 28 1 1 19 10.1016/j.jhealeco.2008.10.007 19058865

[B20] Wei X Liang X Walley J Liu F Dong B Analysis of care-seeking pathways of tuberculosis patients in Guangxi, China, with and without decentralised tuberculosis services International Journal of Tuberculosis & Lung Disease 2009 13 4 514 520 19335959

[B21] Wei X Walley J Liang X Liu F Zhang X Li R Adapting a generic tuberculosis control operational guideline and scaling it up in China: a qualitative case study BMC Public Health 2008 8 260 10.1186/1471-2458-8-260 18662410 PMC2515317

[B22] NCTB National evaluation report on the performance of microscopy centres in township hospitals 2010 Beijing: China National Centre for TB Prevention and Control

[B23] Bernatas JJ Ali IM Ismael HA Matan AB Aboubakar IH Decentralisation of directly observed treatment in a large African city: evaluation of the experience of Djibouti International Journal of Tuberculosis & Lung Disease 2003 7 8 724 729 12921147

[B24] Drabo KM Dauby C Coste T Dembele M Hien C Ouedraogo A Macq J Ouedraogo JB Dujardin B Decentralising tuberculosis case management in two districts of Burkina Faso International Journal of Tuberculosis & Lung Disease 2006 10 1 93 98 16466044

[B25] El-Sony AI Mustafa SA Khamis AH Enarson DA Baraka OZ Bjune G The effect of decentralisation on tuberculosis services in three states of Sudan International Journal of Tuberculosis & Lung Disease 2003 7 5 445 450 12757045

[B26] Kangangi JK Kibuga D Muli J Maher D Billo N N'Gang'a L Ngugi E Kimani V Decentralisation of tuberculosis treatment from the main hospitals to the peripheral health units and in the community within Machakos district, Kenya International Journal of Tuberculosis & Lung Disease 2003 7 9 Suppl 1 S5 13 12971649

[B27] Newell JN Collins CD Baral SC Omar MA Pande SB Decentralisation and TB control in Nepal: understanding the views of TB control staff Health Policy 2005 73 2 212 227 10.1016/j.healthpol.2004.11.014 15978964

[B28] Nyirenda TE Harries AD Gausi F van Gorkom J Maher D Floyd K Salaniponi FML Decentralisation of tuberculosis services in an urban setting, Lilongwe, Malawi International Journal of Tuberculosis & Lung Disease 2003 7 9 Suppl 1 S21 28 12971651

[B29] Harries AD A K Schoevers MA Boeree MJ Nunn P Salaniponi FM Nyangulu DS Case finding for pulmonary tuberculosis, Queen Elizabeth Central Hospital, Blantyre, Malawi The International Journal of Tuberculosis and Lung Disease 1997 1 6 523 527 9487450

[B30] Uplekar M The STOP TB Strategy: Building on and enhancing DOTS to meet the TB related Millennium Development Goals Geneva 2006 15

