# Supplementary material for: Evaluating the impact of decentralising tuberculosis microscopy services to rural township hospitals in gansu province, china
Source: BMC Public Health. 2011 Feb 15;11:103. doi: 10.1186/1471-2458-11-103 (PMC3048529; doi:10.1186/1471-2458-11-103)
Supplement: Additional File 1 — Questionnaire for suspected pulmonary tuberculosis cases of the study. As requested by the editor, the patient questionnaire used in this study was translated into English and presented for readers. Use of the questionnaire for research purpose is encouraged. However, any use of it should be noticed to the authors and must be properly cited in any related research products. [file 1471-2458-11-103-S1.DOC]

Questions:

We appreciate your participation in this survey. The main objective of the survey is to understand your health seeking behavior when you get TB. The result will help to improve the TB control in the future and provide better service to TB cases. There are no standard answers to these questions. Please answer these questions based on your own opinions and experiences. All survey results are anonymous, and your personal information will not be revealed in the report. It takes about half an hour to finish this survey; however, you can quit the survey and require breaking any time. Do you agree to participate in the survey? (If Yes, please ask the patient to sign or approve the Inform Consent Form separately).

ID. of Questionnaire：___________

ID of the Patient：___________

Home Add ：_______Province_______County_______ Township_______Village

**1. General information**

1.1 Sex： 1） M 2） F

1.2 Date：_______/ _______(yyyy/mm)

1.3 Education level

1）Never been to school 2）Primary school 3）Junior middle school

4）Senior middle school 5）Junior college 6）undergraduate or above

1.4 Marital status：

1） Unmarried 2） Married and spouse is still living 3） widowed 4）Divorced

1.5 Job

1） Peasant 2) Worker 3）Migrant worker

4） Businessman 5） Student 6） Others（please specify：_______）

1.6 Geographical features of residence：

1）Plain 2）Hills 3）Mountain area 4）Island

5）Others（please specify：_______）

1.7 No of family member： _______persons（Family members means members of the household living in a same place with economic accounting together）

1.8 What are your annual expenses of your family? _______ Yuan

Net income _______ Yuan，with main source of income from（single selection）:

1）Yourself 2）Other family members 3）Yourself and other family members

1.9 How far from your home to the county centre?

1.10 The most frequently used transportation to the county centre（Single selection）：

1）Bus 2）Bicycle 3）Motorcycle 4）Tractor(tricycle)

5）On foot 6）Others（please specify：_______）

1.11 Return time and cost by the most frequently used transportation to the county centre? _______minutes， _______Yuan

1.12 How far from your home to the township centre?

1.13 The most frequently used transportation to the township centre（Single selection）：

1）Bus 2）Bicycle 3）Motorcycle 4）Tractor(tricycle)

5）On foot 6）Others（please specify）：_______）

1.14 Return time and cost by the most frequently used transportation to the county centre? _______minutes， _______Yuan

1.15 Have you joined the New Rural Medical Cooperation Scheme?

1）Yes 2）No

**2. Health service seeking behavior of the TB suspects**

2.1 Diagnosis classification of the patient（elicited from Outpatient Register and TB Patient Register of county TB dispensary）：

1）Suspected pulmonary TB case 2）New smear positive TB case 3）Smear positive retreatment TB case 4）Smear negative TB case 5）Extrapulmonary TB（please specify site_______）

2.2 When did you get the initial diagnosis of PTB：_______/_____/____(yyyy/mm/dd)

Institute of the initial diagnosis of PTB：

1）Township hospital 2） General hospital at county level or above

3）Hospital of Chinese medicine at county level or above 4）County TB dispensary 5）TB special hospital

6）Others（please specify）：_______

2.3 What were your earliest TB symptoms:（multiple choices）

1）Cough 2）Expectoration 3）Night sweat 4）Hemoptysis 5) Afternoon low-grade fever 6）Hypodynamia or emaciated

7）Chest distress 8）Chest pain 9 Others（please specify）:_______

2.4 When did the earliest TB symptoms emerge_______/_____/____(yyyy/mm/dd)

2.5 When did you visit the doctor for the first time since the onset of symptoms_______/_____/____(yyyy/mm/dd)

2.6 What was the health facility your first visited ：

1）Village clinic 2）Township hospital

3） General hospital at county level or above

4）Hospital of Chinese medicine at county level or above

5）County TB dispensary 6）TB special hospital 7）Others（please specify：

2.6a If it was village clinic, did the village doctor recommend you to higher level facilities?

1）Yes 2）No

2.6b.If yes，what kind of institute did the village doctor recommend?

1）Township hospital 2） General hospital at county level or above

3）Hospital of Chinese medicine at county level or above 4）County TB dispensary 5）TB special hospital

6）Others（please specify）：_______

2.7 Before you visited the county TB dispensary, did you know township hospital where you were from could conduct TB test?

1）Yes 2）No

2.7a. If yes, how did you know that?

1）publicity of Township hospital 2）Publicity material by county TB dispensary

3）Relatives and friends 4）Newspaper 5）Others（please specify：_______

2.8 Have you been to the township hospital for TB test before you came here?

1）Yes 2）No(to question 9)

2.8a. If yes, why（multiple choice，3 at maximum）：

1）Convenient traffic and time-saving 2）Inexpensive 3）Higher reimbursement proportion from health insurance 4）No time to the county centre 5）Felt no need to go to county and above hospital due to the light symptoms 6）Having friends working in the township hospital

7）Others（please specify：_______）

2.8b. What examinations did you get in the township hospital（multi-select）？

1）Doctor consultation 2）Chest radiograph 3）Chest X-ray

4）Sputum smear 5）Others（please specify：_______

2.8c. How much did you spend in township hospital for TB? _____Yuan

2.8d What was the result of the sputum smear in the township hospital：

1）No tubercle bacillus detected

2）Tubercle bacillus detected

3）Did not receive sputum examination

2.8e Before you went to township hospital, did you have any access to the TB knowledge?

1）Yes 2）No

2.8f .If yes, source of information (multiple choice)：

1）Newspaper and magazine 2）Broadcast, film, TV and video 3）School 4）Health publicity（e.g. poster, leaflet, health exhibition） 5）Relatives and friends 6）consultation from doctors 7）Others（please specify）:

2.9. If you had not visited the township hospital before visiting the county TB dispensary, but the general hospital at county level and above, what is the reason(multiple choice)?

1）Low technical capacity of the township hospital 2）Poor facilities in the township hospital 3）County level is more reliable since I have serous symptoms 4）More convenient traffic to the county level 5）Having friends in county level 6）Others（please specify）：_______）

2.9a Did you any access to TB information before visiting the county TB dispensary?

1）Yes 2）No

2.9b.If yes, source of information (multi-select)：

1）Newspaper and magazine 2）Broadcast, film, TV and video 3）School 4）Health publicity（e.g. poster, leaflet, health exhibition） 5）Relatives and friends 6）consultation from doctors 7）Others（please specify）：

2.10. Flow of health seeking behavior of suspected PTB cases：

| **No. of consulting** | **Health facility visited** | **Distance between residence to the health facility（km）** | **Means of transportation **** | **Return transportation cost（Yuan）** | **Test items（ “√”）** | | | **Diagnostic result***** | **Medical expenses（Yuan）** | **Food and Accommodation（Yuan）** |
| --- | --- | --- | --- | --- | --- | --- | --- | --- | --- | --- |
|  |  |  |  |  | **Sputum smear** | **X-ray** | **CT** |  |  |  |
| 1st |  |  |  |  |  |  |  |  |  |  |
| 2nd |  |  |  |  |  |  |  |  |  |  |
| 3rd |  |  |  |  |  |  |  |  |  |  |
| 4th |  |  |  |  |  |  |  |  |  |  |
| 5th |  |  |  |  |  |  |  |  |  |  |
| * Institute of consulting：1 village clinic，2 township hospital， 3 general hospital at county level and above， 4 hospital of Chinese medicine at county level and above， 5 county TB dispensary 6 TB special hospital | | | | | | | | | | |
| **Means of transportation：1 bus， 2 bicycle， 3 motorcycle， 4 tractor(tricycle)， 5 on foot | | | | | | | | | | |
| ***Diagnostic result：1 TB， 2 infection of the upper respiratory tract， 3 chronic bronchitis， 4 pneumonia， 5 lung cancer， 6 asthma， 7 others | | | | | | | | | | |

Name of investigator______________________ Date of investigation：_____/_____/____(yyyy/mm/dd)
